# Numerous lymph node metastases in early gastric cancer without preoperatively enlarged lymph nodes: a case report

**DOI:** 10.1186/s40792-020-0795-2

**Published:** 2020-01-30

**Authors:** Chikanori Tsutsumi, Taiki Moriyama, Kenoki Ohuchida, Koji Shindo, Shuntaro Nagai, Reiko Yoneda, Minako Fujiwara, Yoshinao Oda, Masafumi Nakamura

**Affiliations:** 1 Department of Surgery and Oncology, Graduate School of Medical Sciences, Kyushu University, 3-1-1 Maidashi, Higashi-ku, Fukuoka, 812-8582 Japan; 2 Department of Diagnostic and Therapeutic Endoscopy, Kyushu University Hospital, Fukuoka, Japan; 3 Department of Anatomic Pathology, Pathological Sciences, Graduate School of Medical Sciences, Kyushu University, Fukuoka, Japan

**Keywords:** Early gastric cancer, Lymph node metastasis, Proximal gastrectomy

## Abstract

**Background:**

According to the 2018 Japanese gastric cancer treatment guidelines (ver. 5), a reduced extent of lymphadenectomy (D1 or D1+) is indicated for cT1 N0 tumors that do not meet the criteria for endoscopic resection. However, early gastric cancer with multiple lymph node metastases is not unknown, and cases have been reported. We report a case of a patient with early gastric cancer and numerous nodal metastases who underwent laparoscopic proximal gastrectomy based on a preoperative diagnosis of T1 N0.

**Case presentation:**

A 69-year-old woman underwent emergent endoscopic hemostasis for massive hematemesis of the stomach, and endoscopic examination showed ulceration with a visible vessel. Pathological biopsy examination of the ulcer identified poorly differentiated adenocarcinoma with signet ring cells. The patient was diagnosed with early gastric cancer that was not indicated for endoscopic resection because of the ulceration and histological type. Endoscopic ultrasound showed that the third layer was poorly demarcated at the ulcer scar, indicating invasion to the submucosal layer. Computed tomography did not reveal enlarged lymph nodes or distant metastasis. The preoperative diagnosis was early gastric cancer of the fundus without nodal metastasis, and laparoscopic proximal gastrectomy with D1+ lymphadenectomy was performed. The initial postoperative pathological diagnosis was intramucosal carcinoma without lymphovascular invasion; however, the presence of 26 lymph node metastases was revealed unexpectedly. Additional pathological examination of more resected specimens transected every 2–3 mm revealed that only one lesion contained a small number of cancer cells in the lymphatic duct below the muscularis mucosa.

**Conclusions:**

We report a case of early gastric cancer with 26 nodal metastases in which lymph node involvement was not identified prior to surgery. These findings indicate that the extent of lymphadenectomy and the surgical procedure should be carefully decided even in cT1 N0 early gastric cancer when several risk factors for lymph node metastasis are present.

## Background

Surgery is the most appropriate treatment for patients with early gastric cancer in which endoscopic resection is not indicated. In Japan, the number of laparoscopic surgeries for gastric cancer is increasing annually [1]. According to the Japanese gastric cancer treatment guidelines (ver. 5; 2018), D1 or D1+ lymphadenectomy is indicated for cT1 N0 tumors that do not meet the criteria for endoscopic mucosal resection/endoscopic submucosal dissection, whereas D2 lymphadenectomy is indicated for T2–T4 tumors, as well as for cT1N+ tumors. Although lymph node metastasis is an important prognostic factor in early gastric cancer, its preoperative and/or intraoperative diagnosis remains unreliable. Therefore, Japanese gastric cancer treatment guidelines recommend that D2 lymphadenectomy be performed even in patients with early gastric cancer when preoperative imaging shows any indication of nodal involvement. However, we experienced a case of early gastric cancer with numerous pathological lymph node metastases (pN3b), without nodal involvement in preoperative imaging (cN0). Although cases of early gastric cancer with multiple lymph node metastases are not unknown, few reports to date have described this pathology [2–4]. We report a patient with cT1 N0 early gastric cancer with 26 lymph node metastases, in which laparoscopic proximal gastrectomy with D1+ lymphadenectomy was performed based on the preoperative imaging diagnosis and intraoperative findings of no nodal metastasis.

## Case presentation

**Fig. 1:**
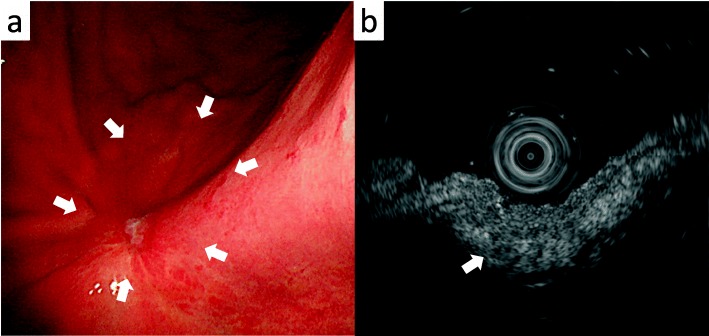
Endoscopic findings. **a** Endoscopy image showing a type 0–IIc lesion with an ulcer scar on the greater curvature side of the fundus (arrow). **b** Endoscopic ultrasound showing a poorly demarcated third layer at the ulcer scar (arrow)

**Fig. 2:**
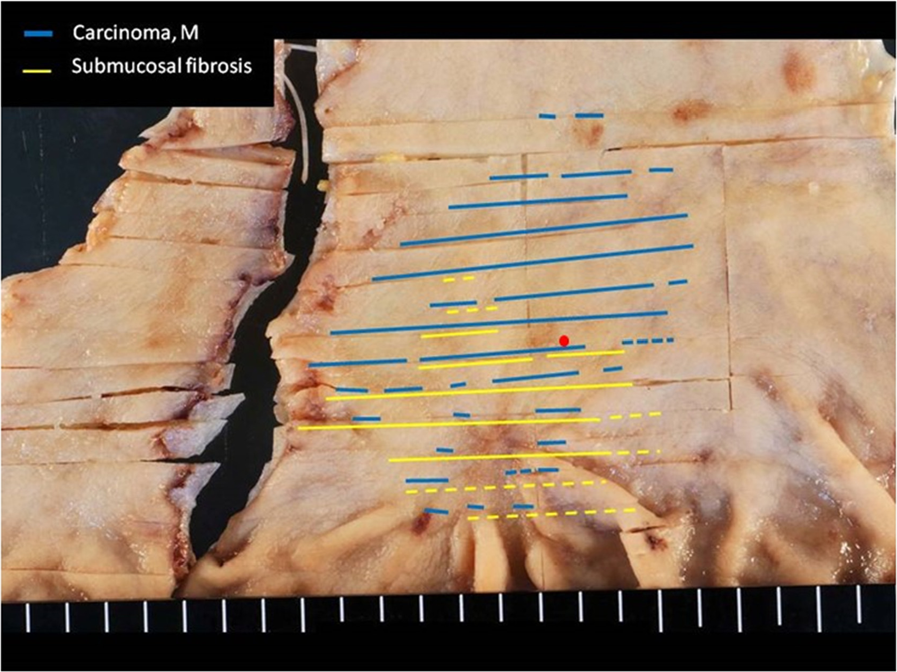
Tumor mapping of the resected specimen. The carcinoma and fibrosis overlapped partially. Lymphatic invasion below the muscularis mucosa was identified at the position of the red dot

**Fig. 3:**
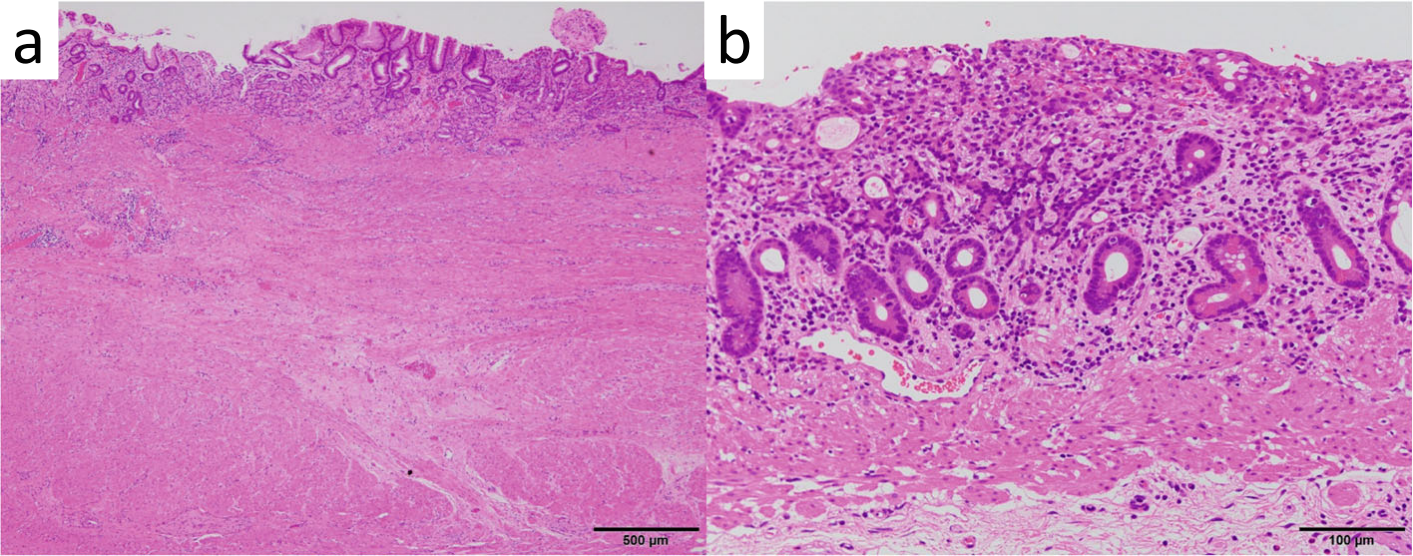
Histopathological findings. Initial pathology showing intramucosal carcinoma without lymphatic and vascular invasion, and with fibrosis affecting all layers. **a** Hematoxylin-eosin staining, original magnification × 40. **b** Hematoxylin-eosin staining, original magnification × 200

**Fig. 4:**
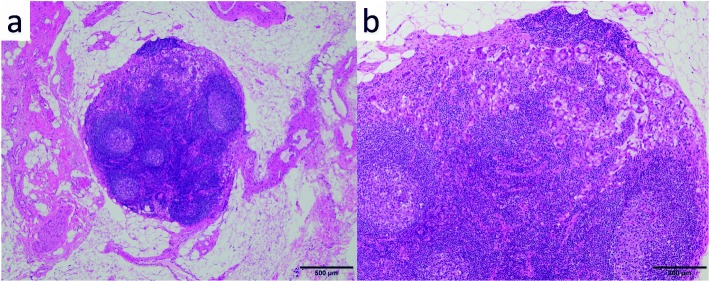
Histopathological findings. Lymph node metastases from the primary lesion. **a** × 40 and **b** × 100 magnification

**Fig. 5:**
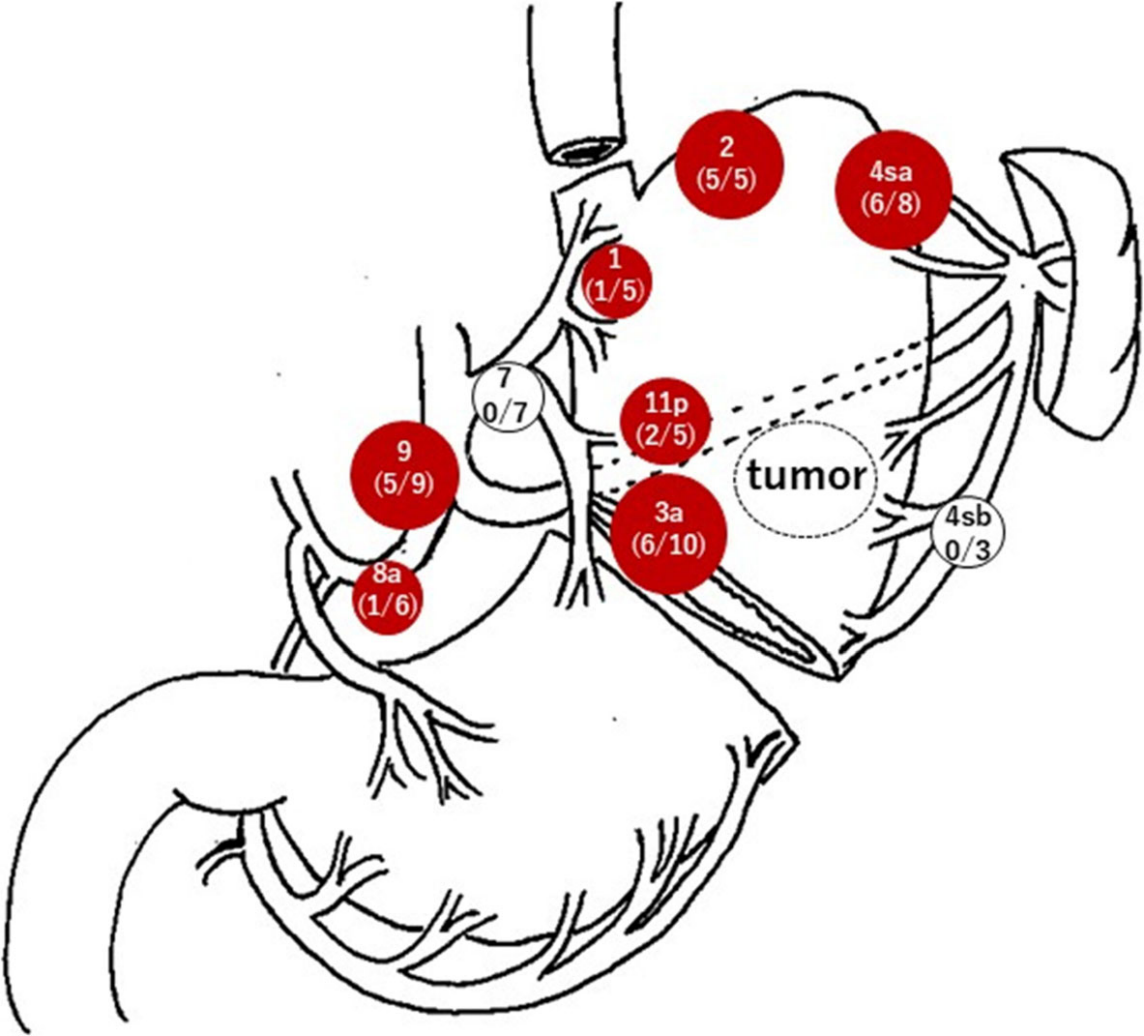
Lymph node mapping. Lymph node metastasis was widely seen in almost all lymph node stations except for stations #4sb and #7 (red circle)

**Fig. 6:**
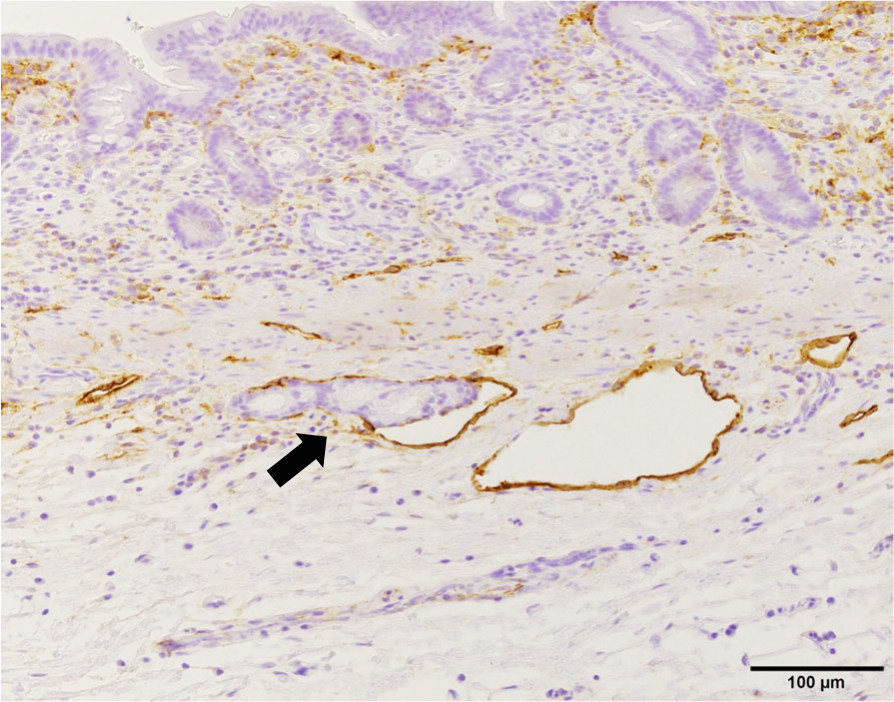
Pathological findings in the additional examined sample showing lymphatic invasion below the muscularis mucosa with D2-40 staining; original magnification × 200

A 69-year-old woman underwent emergency endoscopic hemostasis at her local hospital for massive bleeding of the stomach. Endoscopic examination revealed an irregular ulcerative lesion with a visible vessel. Histopathological examination of a biopsy specimen of the ulcer confirmed the presence of poorly differentiated adenocarcinoma with signet ring cells. The patient was diagnosed with early gastric cancer that did not meet the criteria for endoscopic resection because of its ulceration and histological type, and she was subsequently referred to our hospital for surgical treatment. Physical examination and laboratory data, including tumor markers, revealed no specific findings. The patient also had no relevant medical history. Following treatment for gastric ulcer hemorrhage, the lesion was clarified as type 0–IIc with an ulcer scar 35 mm in size on the greater curvature side of the fundus (Fig. 1a). Endoscopic ultrasound revealed that the third layer was poorly demarcated at the ulcer scar, indicating invasion of the submucosal layer (Fig. 1b). Computed tomography (CT) did not reveal enlarged lymph nodes or distant metastasis. Following a clinical diagnosis of early gastric cancer confined to the submucosa (T1b) without lymph node metastasis and with intraoperative findings of no nodal metastasis, laparoscopic proximal gastrectomy with D1+ lymphadenectomy was performed. An additional resection of the distal side of the stomach was performed because the tumor was close to the resection margin. The operating time was 257 min, and the blood loss volume, including ascites, was 120 mL. Postoperative pathological examination revealed poorly differentiated adenocarcinoma with signet ring cells, 49 × 44 mm in size, with abundant fibrosis affecting all layers (Figs. 2 and 3). The initial pathological diagnosis was intramucosal carcinoma (T1a) without lymphovascular invasion (Ly0, V0). However, 26/58 lymph nodes showed metastasis (N3b); n#1 (1/5), n#2 (5/5), n#3a (6/10), n#4sb (0/3), n#4sa (6/8), n#7 (0/7), n#8a (1/6), n#9 (5/9), and n#11p (2/5) (Figs. 4 and 5). Further pathological examination was performed with more resected specimens transected every 2–3 mm and with D2-40 staining. The re-examined pathological samples identified only one lesion containing a small number of cancer cells in the lymphatic duct below the muscularis mucosa (Fig. 6). Therefore, the patient was diagnosed as having T1b gastric cancer with ly1 because of the single lesion in the lymphatic duct, although direct invasion to the submucosal layer was not found, even with further examination. The final pathological diagnosis was T1b gastric cancer with multiple lymph node metastases (pT1bN3bM0, pStage IIB). Postoperatively, the patient followed an uneventful course with no complications. We planned remnant gastrectomy and additional lymph node dissection, but the patient elected not to undergo the additional surgical treatment and selected only adjuvant chemotherapy with capecitabine/oxaliplatin. To date, there has been no evidence of recurrence more than 12 months after surgery.

## Discussion

The frequency of lymph node metastasis in early gastric cancers has been reported as 8.9% (2.5% in mucosal cancers and 17.6% in submucosal cancers) [﻿5﻿]. However, there are no reliable preoperative imaging diagnostic methods to accurately detect lymph node metastasis in early gastric cancer without enlarged lymph nodes.

The findings in the present case indicate that standard gastric resection with D2 lymph node dissection should be considered in patients with early gastric cancer who have several risk factors for lymph node involvement but no findings of nodal metastasis in preoperative imaging. Although a precise assessment of the extent of gastric cancer spread is important prior to surgery to determine the optimum treatment strategy for patients with early-stage disease, preoperative detection of lymph nodes with micrometastases is challenging. The accuracy of multidetector-row CT, [18F]-fluorodeoxyglucose-positron emission tomography (FDG-PET)/CT, and endoscopic ultrasound for detecting lymph node metastasis has been reported as 83%, 68%, and 81%, respectively [﻿6﻿, 7, 8]. In addition, a retrospective study reported that the incidence of metastasis was 25% when the lymph node imaged was < 1.0 cm in size [﻿9﻿]. The patient in the present case was preoperatively diagnosed as having early gastric cancer without nodal metastasis according to imaging findings. Although laparoscopic proximal gastrectomy with D1+ lymph node dissection was performed according to the Japanese gastric cancer treatment guidelines, 26 lymph nodes were confirmed to have metastases (N3b). Lymph node metastasis was widely seen in almost all lymph node stations including stations #9 and #11p. A previous systematic review and meta-analysis reported that the frequency of metastasis to level 2 lymph nodes was 4.9% in patients with submucosal cancer [10﻿]. Several studies recently reported that large tumor size, macroscopically depressed type, invasion into the muscularis mucosae, lymphatic invasion, intratumoral or ulcer scar, and histologically undifferentiated type are preoperative risk factors for lymph node metastasis in early gastric cancer [﻿5﻿, ﻿11﻿, ﻿﻿﻿12﻿﻿﻿]. In our patient, four of these six risk factors (large tumor size, macroscopically depressed type, ulcer scar, and histologically undifferentiated type) were recognized preoperatively. Together, these reports suggest that the appropriateness of the surgical procedure and lymphadenectomy should be carefully considered in patients with early gastric cancer and a high risk of lymph node metastasis.

Intraoperative sentinel lymph node biopsy (SLNB) using combined dye and radioisotope methods has been reported to be effective in identifying nodal metastasis in early gastric cancer in which the identification rate of lymph nodes and the sensitivity for detecting nodal metastasis was 96–99% [﻿13﻿, 14]. When the intraoperative pathological examination of SLNB yields a positive result, D2 lymph node dissection or additional lymph node dissection should be considered. However, the combination of dye and radioisotopes can be used only in centers equipped to perform this assessment. In a multicenter trial performed by the Japan Clinical Oncology Group, the feasibility and accuracy of diagnosis using SLNB with indocyanine green was evaluated, and the rate of false negatives was found to be 46% [﻿15﻿]. Therefore, there are still difficulties in the introduction and accuracy of SLNB. Although the indication for surgical resection in patients with high numbers of risk factors for lymph node metastasis has been discussed in several previous studies, the appropriate surgical procedures for gastrectomy and lymphadenectomy are not clearly described [﻿5﻿, ﻿11﻿, ﻿﻿12﻿﻿]. The intraoperative pathological examination of multiple lymph nodes with or without SLNB is useful in patients with multiple risk factors for lymph node metastasis in early gastric cancer. Based on the pathological results, conversion to total gastrectomy or to more extended lymph node dissection should be considered.

Several studies have reported the presence of large arteries and lymphatics in the submucosal layer [16]. Our patient had a medical history of endoscopic hemostasis for massive hematemesis, indicating carcinoma invasion below the muscularis mucosae at that time. To the best of our knowledge, no clinical study to date has investigated the association between bleeding and nodal metastasis. Our patient’s previous history of massive bleeding may potentially have increased the likelihood of lymph node involvement in early gastric cancer. Further studies are required to clarify the relationship between massive bleeding and nodal metastasis.

Early gastric cancer (T1a or T1b) with multiple lymph node metastases (N3) is diagnosed as stage IIB or stage IIIB, and adjuvant chemotherapy for stage IIB or stage IIIB gastric cancer is recommended according to the Japanese gastric cancer treatment guidelines. However, the recommendation was based on studies enrolling patients with ≥ T2 gastric cancer [17, 18]. Therefore, adjuvant chemotherapy for early gastric cancer with lymph node metastasis remains a controversial issue although several investigators recommend adjuvant chemotherapy for such patients to avoid tumor recurrence after surgical resection [19–21].

When patients with multiple lymph node involvement undergo insufficient lymph node dissection, remnant total gastrectomy with additional lymph node dissection is a promising treatment. However, the clinical effects of further gastrectomy and lymphadenectomy are unclear. Moreover, complications may occur after remnant total gastrectomy, leading to a delay in beginning chemotherapy. Therefore, early adjuvant chemotherapy without additional surgery is another promising strategy. Our patient elected to undergo adjuvant chemotherapy although we recommended remnant total gastrectomy and additional lymph node dissection.

In the present case, the initial pathological diagnosis was intramucosal carcinoma. However, multiple lymph node metastases were detected. Therefore, the resected specimens were subsequently transected every 2–3 mm and were re-examined. During the re-examination, we found only one lesion containing a small number of cancer cells in the lymphatic duct below the muscularis mucosa. We found no direct invasion to the submucosal layer, even in the additional examination. These findings substantially suggest that our patient was a rare case with mucosal cancer accompanied by multiple lymph node metastases. Such tumors are rare but not unknown in patients with early-stage disease [4]. Given the current lack of appropriate imaging modalities and biomarkers to identify such tumors, further studies including molecular biology are needed to develop appropriate diagnostic methodologies.

## Conclusion

We report a case of early gastric cancer with 26 nodal metastases in which lymph node involvement was not identified prior to surgery. The present case indicates that surgical treatment with additional lymph node dissection should be considered in patients with a high preoperative risk of nodal metastasis even in cT1 N0 early gastric cancer. Intraoperative pathological examination of the dissected lymph nodes may be useful to change the range of lymph node dissection in such patients.

## Data Availability

Not applicable

## Abbreviations

CT: Computed tomography
FDG-PET: [18F]-fluorodeoxyglucose-positron emission tomography
SLNB: Sentinel lymph node biopsy
